# Correction: Distinct neuronal populations contribute to trace conditioning and extinction learning in the hippocampal CA1

**DOI:** 10.7554/eLife.74730

**Published:** 2021-10-19

**Authors:** Rebecca A Mount, Sudiksha Sridhar, Kyle R Hansen, Ali I Mohammed, Moona E Abdulkerim, Robb Kessel, Bobak Nazer, Howard J Gritton, Xue Han

Mount RA, Sridhar S, Hansen KR, Mohammed AI, Abdulkerim M, Kessel R, Nazer B, Gritton HJ, Han X. 2021. RDistinct neuronal populations contribute to trace conditioning and extinction learning in the hippocampal CA1. *eLife* **10**:e56491. doi: 10.7554/eLife.56491.

Published 12, April 2021

We inadvertently used the same bar graph for Jaccard Index in Figure 4Dii as we did for Jaccard Index in Figure 2Eii. We corrected the figure. The text remains unchanged, as it already describes the correct results.

The corrected Figure 4 is shown here:

**Figure 1:**
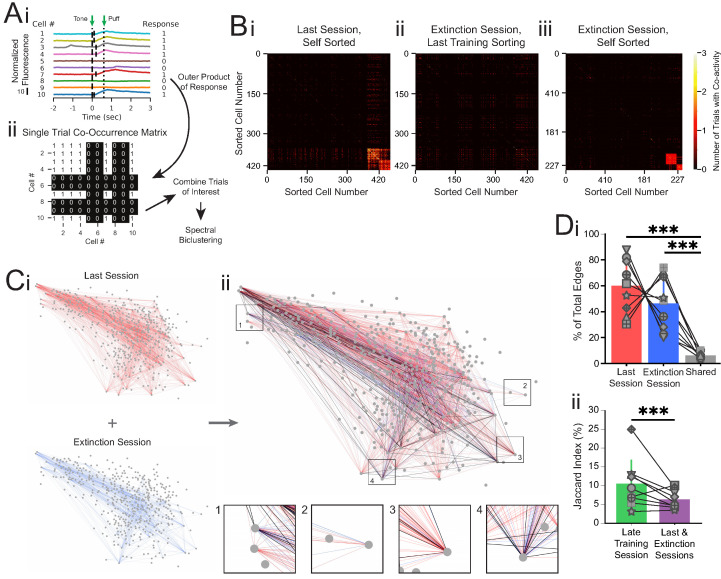


The originally published Figure 4 is shown here for reference:

**Figure 2:**
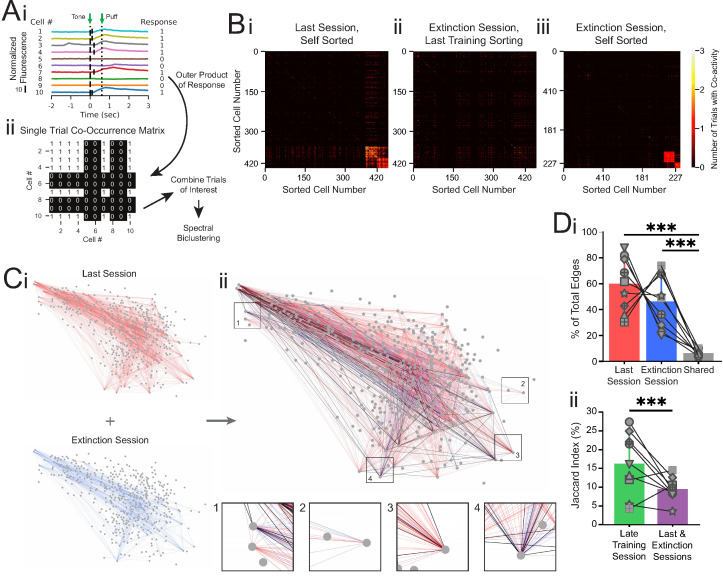


The article has been corrected accordingly.

