# Supplementary material for: Distinct neuronal populations contribute to trace conditioning and extinction learning in the hippocampal CA1
Source: eLife. 2021 Apr 12;10:e56491. doi: 10.7554/eLife.56491 (PMC8064758; doi:10.7554/eLife.56491)
Supplement: Supplementary file 1. [file elife-56491-supp1.docx]

| Mouse | Marker | Number of cells | | |
| --- | --- | --- | --- | --- |
|  |  | First session | Late training | Last session/extinction session |
| 1 | 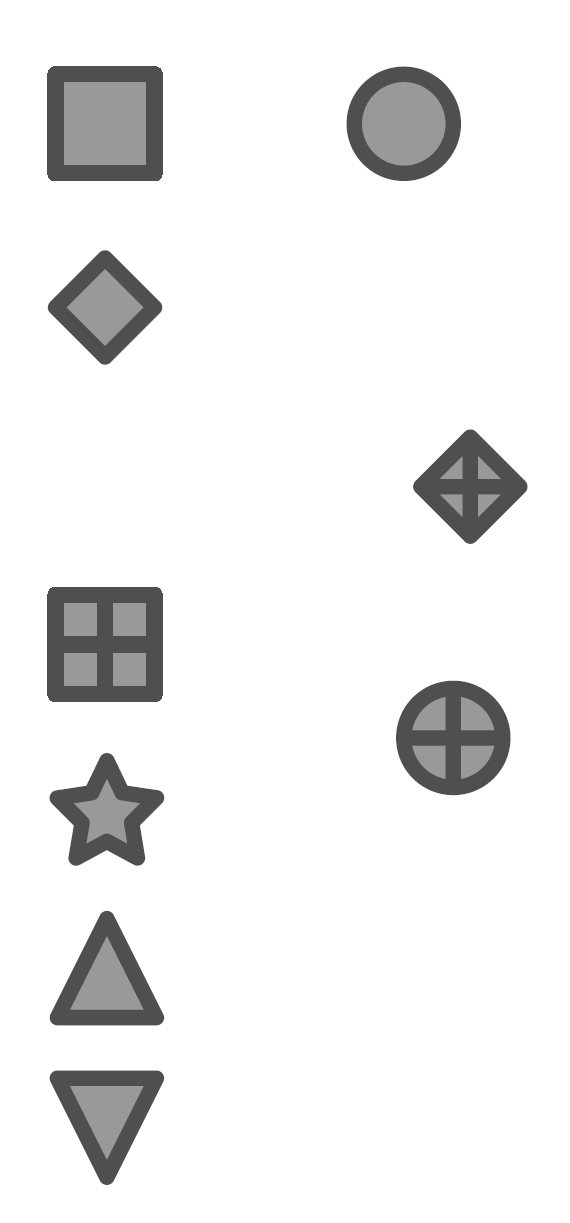 | 862 | 498 | 321 |
| 2 | 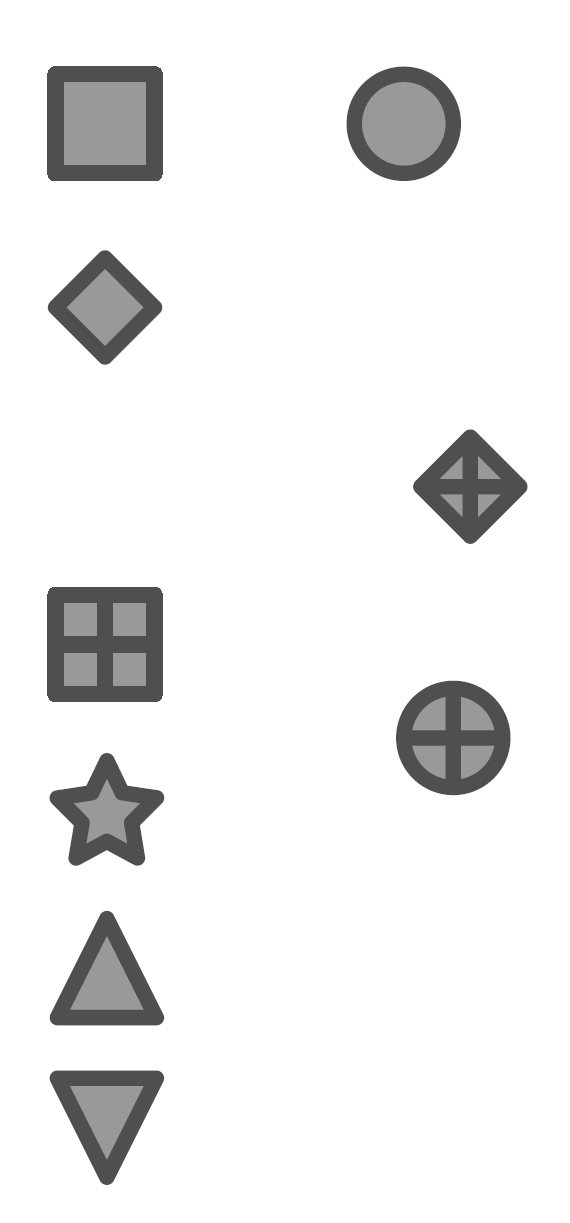 | 761 | 443 | 200 |
| 3 | 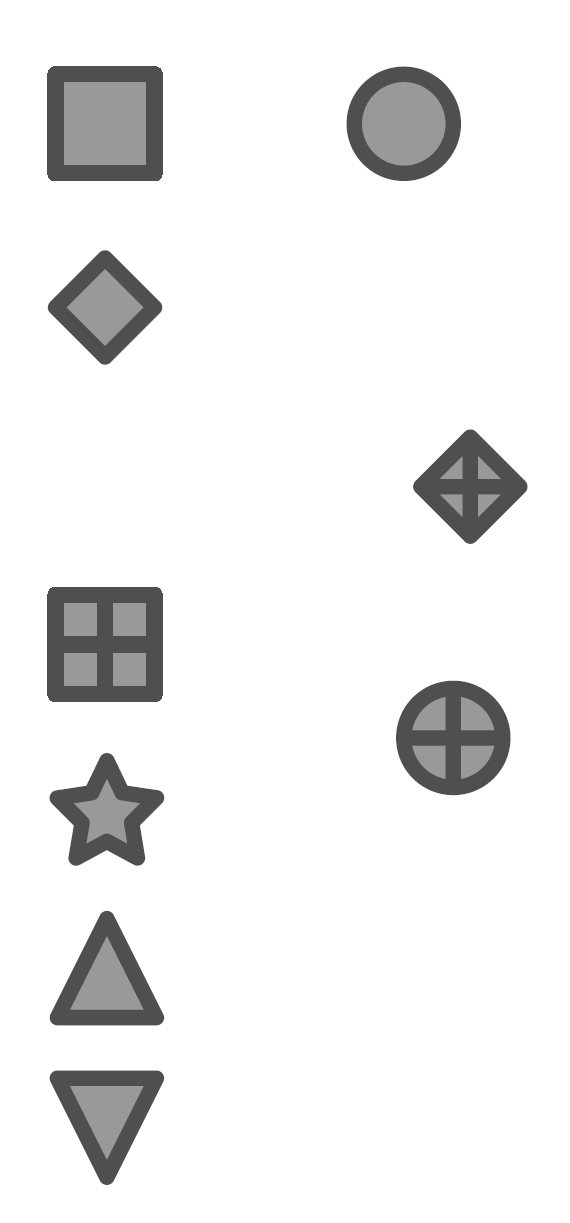 | 756 | 592 | 437 |
| 4 | 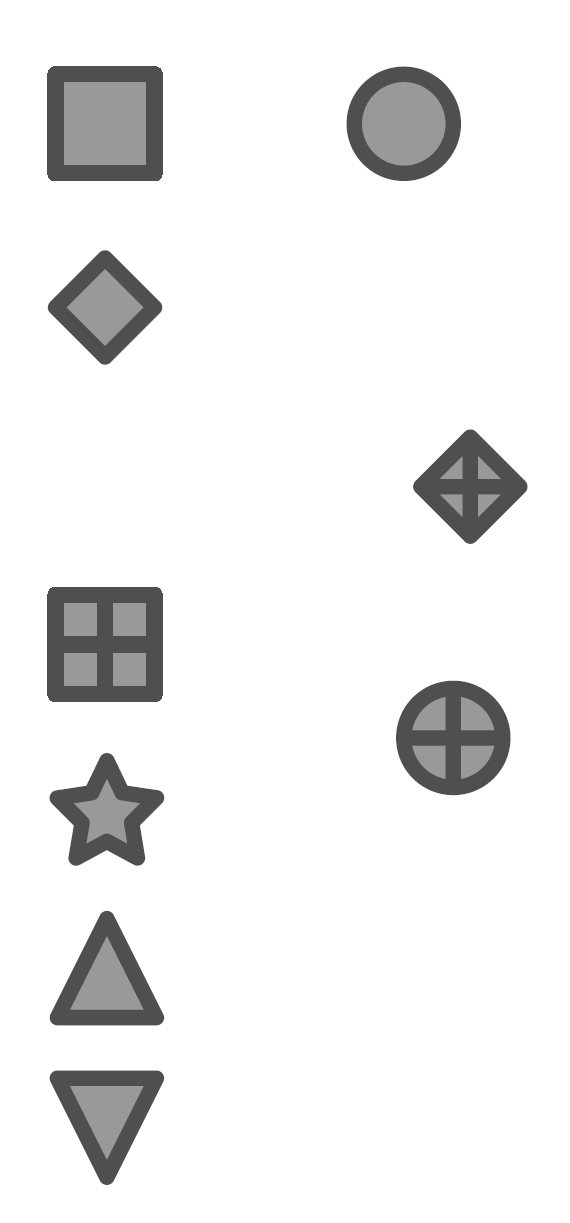 | 115 | 242 | 329 |
| 5 | 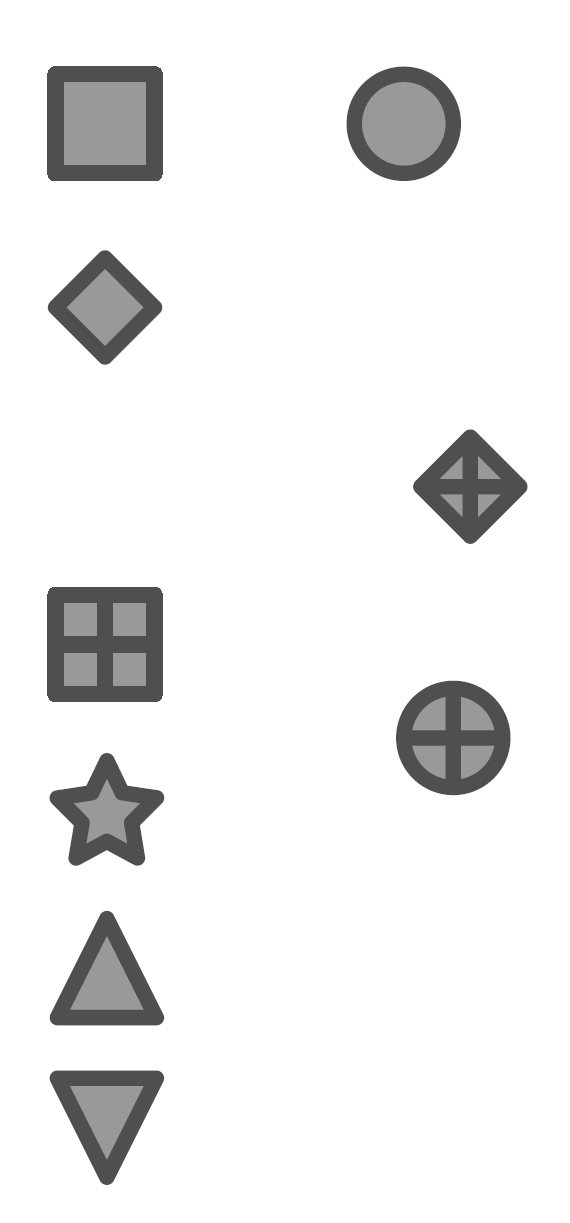 | 187 | 236 | 341 |
| 6 | 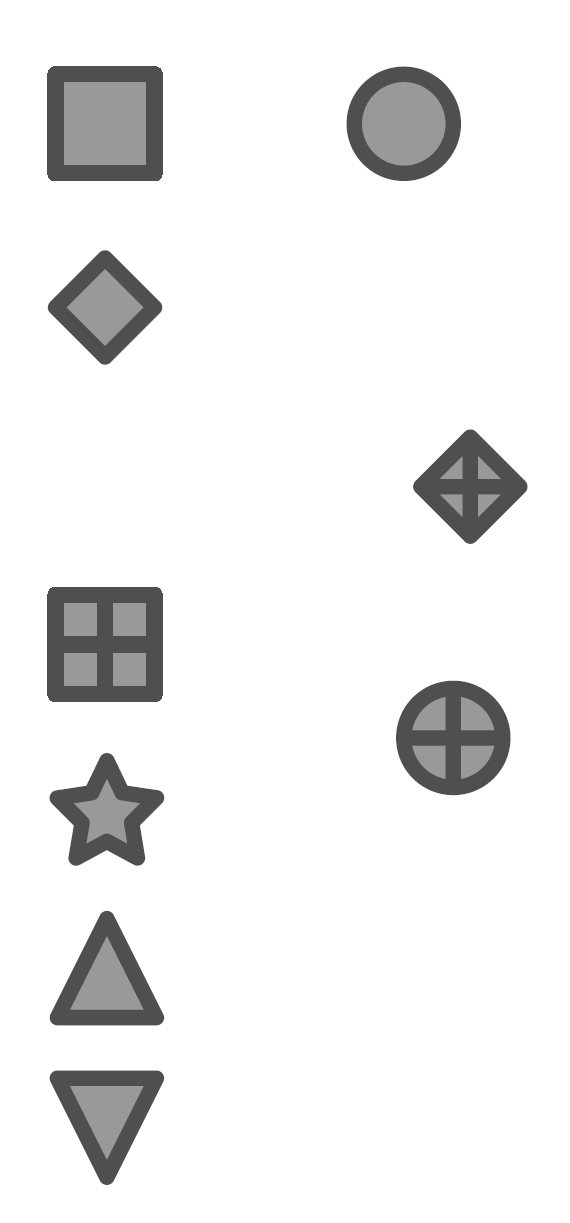 | 113 | 170 | 186 |
| 7 | 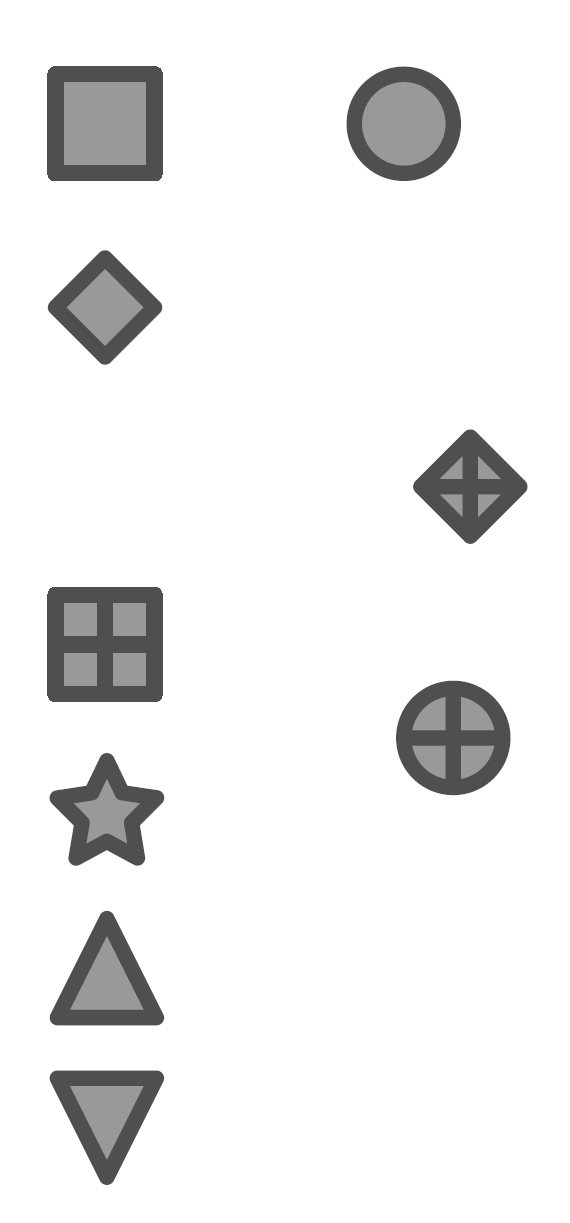 | 143 | 145 | 170 |
| 8 | 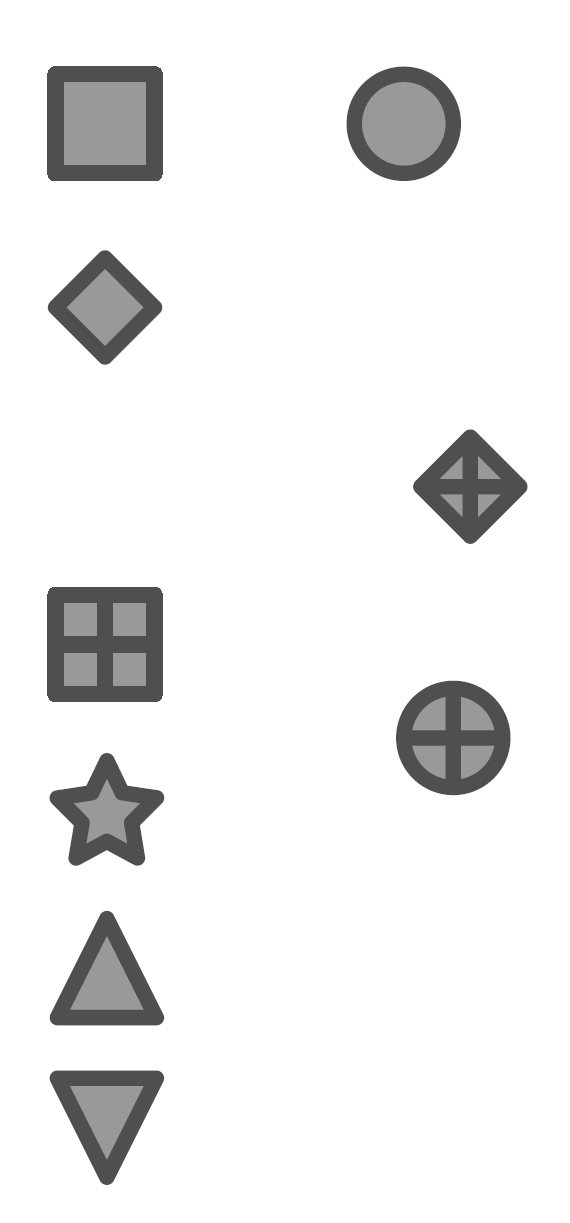 | 234 | 248 | 243 |
| 9 | 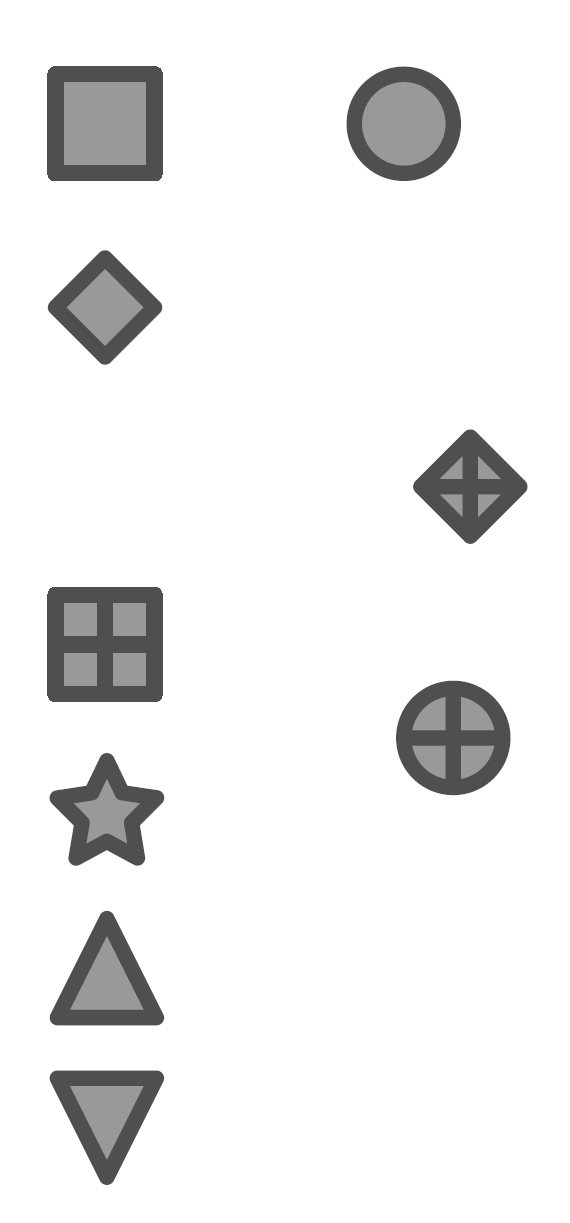 | 70 | 122 | 105 |
| **Total** | | **3241** | **2696** | **2332** |
