# Supplementary material for: Distinct neuronal populations contribute to trace conditioning and extinction learning in the hippocampal CA1
Source: eLife. 2021 Apr 12;10:e56491. doi: 10.7554/eLife.56491 (PMC8064758; doi:10.7554/eLife.56491)
Supplement: Supplementary file 3. [file elife-56491-supp3.docx]

|  | First session | Extinction session |
| --- | --- | --- |
| Responsive cells | 288 (12.17%) | 239 (12.28%) |
| Non-responsive cells | 2079 (87.83%) | 1707 (87.72%) |
