# Supplementary material for: Distinct neuronal populations contribute to trace conditioning and extinction learning in the hippocampal CA1
Source: eLife. 2021 Apr 12;10:e56491. doi: 10.7554/eLife.56491 (PMC8064758; doi:10.7554/eLife.56491)
Supplement: Supplementary file 4. [file elife-56491-supp4.docx]

|  | Last training session | Extinction session |
| --- | --- | --- |
| Responsive cells | 347 (14.88%) | 266 (11.4%) |
| Non-responsive cells | 1985 (85.12%) | 2066 (88.6%) |
