# Supplementary material for: Distinct neuronal populations contribute to trace conditioning and extinction learning in the hippocampal CA1
Source: eLife. 2021 Apr 12;10:e56491. doi: 10.7554/eLife.56491 (PMC8064758; doi:10.7554/eLife.56491)
Supplement: Supplementary file 5. [file elife-56491-supp5.docx]

|  | Late training day | Last training/extinction sessions |
| --- | --- | --- |
| Common responsive cells | 59 (10.65%) | 149 (20.47%) |
| Non-common responsive cells | 495 (89.35%) | 579 (79.53%) |
