# Supplementary material for: Distinct neuronal populations contribute to trace conditioning and extinction learning in the hippocampal CA1
Source: eLife. 2021 Apr 12;10:e56491. doi: 10.7554/eLife.56491 (PMC8064758; doi:10.7554/eLife.56491)
Supplement: Supplementary file 6. [file elife-56491-supp6.docx]

|  | Late training day | Last training/extinction sessions |
| --- | --- | --- |
| Shared edges | 2159 (10.44%) | 600 (8.21%) |
| Non-shared edges | 18517 (89.56%) | 6706 (91.79%) |
